# Assessing Changes in Corneal Densitometry in Patients After Small Incision Lenticule Extraction (SMILE)

**DOI:** 10.7759/cureus.70943

**Published:** 2024-10-06

**Authors:** Shreya Pandya, Muhammed A Jaafar, Kenneth D Han, Garrett N Manion, Kayvon A Moin, Stephanie Zhang, Majid Moshirfar, Phillip C Hoopes

**Affiliations:** 1 Ophthalmology, University of Louisville School of Medicine, Louisville, USA; 2 Ophthalmology, University of Arizona College of Medicine - Phoenix, Phoenix, USA; 3 Ophthalmology, Creighton University School of Medicine, Omaha, USA; 4 Hoopes Vision Research Center, Hoopes Vision, Draper, USA; 5 School of Medicine, American University of the Caribbean, Cupecoy, SXM; 6 John A. Moran Eye Center, University of Utah School of Medicine, Salt Lake City, USA; 7 Eye Banking and Corneal Transplantation, Utah Lions Eye Bank, Murray, USA; 8 Ophthalmology, Hoopes Vision, Draper, USA

**Keywords:** cornea, corneal refractive surgery, eye, haze, lasik, opacity, ophthalmology, pentacam, stroma, visumax

## Abstract

Purpose: The aim is to assess for any lasting changes in corneal densitometry (CD) in patients who underwent small incision lenticule extraction (SMILE) and developed early transient postoperative corneal haze.

Methods: This retrospective chart review analyzed 98 eyes from 49 patients who underwent SMILE at the Hoopes Vision Clinic and had one-year postoperative Pentacam CD (Oculus Optikgeräte GmbH, Wetzlar, Germany). These were compared to 78 eyes from 42 unoperated myopic control patients with documented CD measurements. The preoperative CD, measured in grayscale units (GSU), was compared between SMILE patients who developed early transient haze (“transient haze group”), SMILE patients who did not build haze (“non-haze group”), and patients who did not undergo any refractive surgery (“controls”). The postoperative CD was compared between the transient haze and non-haze groups. Then, the postoperative CD was compared to the preoperative CD for the non-haze group and transient haze group, respectively.

Results: The only significant difference in preoperative CD was in the central layer of the cornea at the 6-10 mm annulus between the non-haze group and controls (17.31 ±3.77 vs. 16.18 ±3.92 GSU; p=0.04). Postoperatively, there were no differences in CD between the non-haze group and the transient haze group (p<0.05). Comparing postoperative CD to preoperative CD, the non-haze group had increased CD in the 2-6 mm annulus of the anterior (1.54 ±0.45 GSU; p=0.001), posterior (0.65 ±2.28 GSU; p=0.032), and full thickness (0.72 ±0.29 GSU; p=0.006) layers of the cornea in addition to an increased CD in the 6-10 mm annulus of the posterior corneal layer (1.19 ±0.55 GSU; p=0.049). The transient haze group had an increased CD in the 2-6 mm annulus of the anterior (2.61 ±1.09 GSU; p=0.021) and full thickness (1.44 ±0.68 GSU; p=0.039) layers of the cornea.

Conclusion: There is no lasting difference in CD between patients who develop postoperative transient haze and those who do not after undergoing SMILE.

## Introduction

Corneal haze is a subepithelial opacification of the cornea that can negatively affect visual quality, often attributed to a pathological healing response following infection, injury, or surgical procedures [1]. Damage to the corneal epithelium triggers the secretion of cytokines and growth factors, such as transforming growth factor β (TGFβ), which promote the development and differentiation of corneal stromal keratocytes into myofibroblasts. Disruption in the regulation of these factors can lead to irregular extracellular matrix (ECM) deposition and excessive production of myofibroblasts [2-4]. These abnormalities in the healing process contribute to the formation of corneal haze, often observed after refractive surgeries involving manipulation of the epithelium [5]. The development of corneal haze postoperatively varies depending on the surgical approach [6]. Subepithelial haze is more commonly observed after surface ablation procedures that disrupt the epithelial basement membrane, such as photorefractive keratectomy (PRK) [7]. In contrast, laser in-situ keratomileusis (LASIK) minimizes epithelial damage, resulting in a lower incidence of postoperative corneal haze [5]. Additionally, in small incision lenticule extraction (SMILE), the creation of only a small peripheral incision likely further reduces the development of postoperative corneal haze [5,8].

Historically, corneal haze has been diagnosed clinically using various grading scales, such as the one proposed by Fantes et al. [9]. However, due to the inherently subjective nature of these scales, several studies have assessed the efficacy of objective measures, such as corneal densitometry (CD) measured by the Pentacam Scheimpflug camera (Oculus Optikgeräte GmbH, Wetzlar, Germany), in predicting haze [10-12]. While previous studies have primarily focused on CD as a predictor of haze following procedures such as PRK and corneal transplantation, to our knowledge, no studies have examined changes in CD values in patients with a history of haze post-SMILE surgery [13]. Therefore, the objective of this study is to assess whether there are any lasting changes in CD in patients with a prior history of early transient haze following SMILE surgery.

## Materials and methods

In this retrospective chart review, we analyzed 98 eyes from 49 patients who underwent SMILE at Hoopes Vision in Draper, UT, between February 2017 and April 2023. All patients who received SMILE and had one-year postoperative Pentacam CD measurements were included. Exclusion criteria were applied to patients without one-year densitometry measurements or those with other potentially confounding ocular pathologies, such as severe dry eye disease, keratopathy, and corneal epithelial irregularities. Each patient underwent thorough pre- and postoperative examinations, including assessments of uncorrected distance visual acuity (UDVA), corrected distance visual acuity (CDVA), Pentacam imaging, and slit-lamp biomicroscopy. Postoperatively, corneal haze was evaluated clinically through slit-lamp biomicroscopy by one corneal refractive surgeon (M.M.) with over 25 years of clinical and surgical experience at three months and one year postoperatively using the grading method described by Fantes et al. Then, the percentage of eyes affected by transient haze with each of these gradings (trace, 1+, 2+, 3+, 4+) was recorded. Additionally, to ensure that SMILE patients had comparable preoperative CD values to non-refractive candidates, we utilized a random number generator to randomly choose 78 eyes from 42 myopic patients who did not undergo any surgical intervention but had CD measurements as our control. The study was approved by the Biomedical Research Alliance of New York institutional review board IRB # A20-12-547-823, adhered to the tenets of the Declaration of Helsinki, and HIPAA regulations were followed. The study was also approved by the Hoopes Vision Ethics Board and the patient’s informed consent was obtained.

CD measurements

Objective grading of corneal clarity was performed using CD measurements obtained from a Scheimpflug camera (Pentacam HR). This device measures the amount of backscattered light in multiple regions of the cornea, expressed in grayscale units (GSU). The GSU scale ranges from 0 to 100, with zero indicating minimal light scatter (maximum transparency) and 100 indicating maximum light scatter (minimum transparency). CD measurements were recorded from the anterior (anterior 120 µm, representing the anterior stroma), central, posterior (posterior 60 µm, consisting of the deep posterior stroma, Descemet’s membrane, and endothelial layer), and total corneal layers. Measurements from each anatomical layer were taken from four concentric annuli (0-2 mm, 2-6 mm, 6-10 mm, and 10-12 mm) surrounding the corneal apex.

Surgical procedure

A VisuMax 500 kHz femtosecond laser system (Carl Zeiss Meditec, Jena, Germany) was used to perform SMILE. For all patients, the cap thickness and diameter were set at 120 µm and 7.5 mm, respectively. The laser energy was set at 125 nJ, with a spot distance of 3.70 µm for the lenticule, 3.80 µm for the cap, and 2.00 µm for both the lenticule side cut and the cap side. For myopic correction, the lenticule diameter was 6.5 mm, ranging from 6.0 to 6.5 mm for myopic astigmatism correction. A 3.0 mm incision was made, and the lenticule was extracted using a blunt spatula. Postoperatively, patients followed a regimen of moxifloxacin 0.5% ophthalmic solution and prednisolone acetate 1% ophthalmic suspension, administered four times daily (QID) for one week, then the prednisolone acetate 1% ophthalmic suspension was given twice daily (BID) for two weeks.

Statistical analysis

Microsoft Excel (ver. 16.0; Microsoft Corporation, Redmond, WA), IBM Statistical Package for Social Sciences (SPSS ver. 29.0; IBM Corp., Armonk, NY), and G*Power (version 3.1, Düsseldorf, Germany) were used for data collection and statistical analysis. The normality of each dataset was assessed using the Shapiro-Wilk test. Preoperative CD measurements were compared across three patient groups - non-haze, transient haze, and control - for each corneal layer (anterior, central, posterior, and total) and concentric annulus (0-2 mm, 2-6 mm, 6-10 mm, and 10-12 mm). Postoperative measurements for the three groups were assessed similarly.

Within the non-haze and transient haze groups, preoperative and postoperative measurements were compared to evaluate changes over the year following surgery. Paired samples with parametric data were analyzed using the paired t-test, while non-parametric data were analyzed using the Wilcoxon signed-rank test. For unpaired samples, parametric data were assessed using the unpaired t-test, and non-parametric data were analyzed with the Mann-Whitney test. Statistical significance was set at an alpha level of 0.05, and the least significant difference analysis was performed to adjust for multiple comparisons. The statistical power of the unpaired t-test was evaluated using G*Power software. For our α value of 0.05, a medium effect size of 0.5, and an allocation ratio of 1.25, a sample size of 102 eyes was needed to achieve a statistical power of 0.80. Additionally, a post hoc analysis of the unpaired t-test of our sample size of 176 eyes (98 eyes with transient haze + 78 eyes without haze) revealed a statistical power of 0.95.

## Results

Preoperative characteristics

As mentioned earlier, this retrospective chart review analyzed 49 patients (98 eyes) who underwent SMILE at a single-site refractive surgery center in Draper, UT, between February 2017 and April 2023. Of these, 11 patients (17 eyes) had developed corneal haze during the three-month postoperative period, resulting in an occurrence rate of 17%. This included 14 eyes with trace haze and three eyes with 1+ haze. There were no eyes with 2+, 3+, or 4+ haze documented. At the one-year postoperative period, all cases of haze had resolved except for two eyes with trace haze.

Preoperatively, patients undergoing SMILE on average were younger (36.0 ± 7.1 years vs. 42.3 ± 7.9 years; p<0.01) had a more myopic sphere (-5.11 ± 1.40 D vs. -2.83 ± 2.56 D; p=0.01), less cylindrical astigmatism (-0.49 ± 0.45 D vs -0.71 ± 0.70 D; p<0.01), more myopic SEQ (-5.36 ± 1.40 D vs. -3.19 ± 2.54 D; p<0.01), and thicker corneas (554.83 ±28.77 µm vs. 539.44 ± 30.41 µm; p<0.01) compared to controls. Otherwise, there were no statistically significant differences in sex (42.1% male/57.9% female vs. 47.5% male/52.5% female; p>0.05), K1 (43.4 ± 1.0 D vs. 43.7 ± 1.4 D; p>0.05), or K2 (44.3 ± 1.1 D vs. 44.8 ± 1.2 D; p>0.05) between SMILE patients and controls (Table 1).

**Table 1:** Preoperative patient demographics (Control vs all SMILE) Values are provided as mean ± standard deviation. For sex, values are provided as a percentage of the overall cohort. Statistical significance determined with two sample T-tests, set at p<0.05. Abbreviations: SMILE: small incision lenticule extraction, D: diopter, SEQ: spherical equivalent, and CCT: central corneal thickness.

Parameter	Control	Range	All SMILE	Range	T-value	P-value
Age (years)	42.3 ± 7.9	(26 to 62)	36.0 ± 7.1	(23 to 56)	5.967	<0.01
Sex (male%/female%)	38/42	(47.5/52.5)	40/55	(42.1/57.9)	0.799	0.477
Sphere (D)	-2.83 ± 2.56	(-13.0 to 3.50)	-5.11 ± 1.40	(-8.50 to -2.25)	4.824	<0.01
Cylinder (D)	-0.71 ± 0.70	(-3.0 to 0.20)	-0.49 ± 0.45	(-1.75 to 0.00)	5.192	<0.01
SEQ (D)	-3.19 ± 2.54	(-13.5 to 2.89)	-5.36 ± 1.40	(-8.50 to -2.75)	4.678	<0.01
K1 (D)	43.7 ± 1.4	(40.2 to 47.7)	43.4 ± 1.0	(41.7 to 45.8)	0.543	0.995
K2 (D)	44.8 ± 1.2	(42.1 to 47.9)	44.3 ± 1.1	(42.3 to 47.3)	0.496	0.232
CCT (µm)	539.44 ± 30.41	(470 to 621)	554.83 ± 28.77	(496 to 616)	4.354	<0.01

Preoperative CD measurements were also compared among the control, non-haze, and transient groups. The non-haze group showed significantly higher CD at the 6-10 mm annulus of the central corneal layer compared to the control group (17.31 ± 3.77 GSU vs. 16.18 ± 3.92 GSU; p=0.04). However, no other significant differences in preoperative densitometry measurements were observed among the three groups (Tables 2-5).

**Table 2:** Corneal densitometry comparisons of the anterior corneal layer “Preop-Postop” section indicates magnitude of change from preoperative to postoperative measurements. Comparisons between study groups and control group for “Postop” not available, as control group patients did not undergo surgery. Statistical significance for “Preop” section determined with one-way ANOVA (F score) and “Postop” and “Preop-Postop” section determined with two-sample T-test (T-value); set at p<0.05. Values represented mean ± standard deviation; Units = grayscale units (GSU) * Indicates statistically significant increase in corneal densitometry from “Preop” to “Postop” within the specified group (p<0.05).

Annulus (mm)	Non-Haze Group (N=81)	Transient Haze Group (N=17)	Controls (N=78)	F score/T-value	P-value
Preop					
0-2	25.26 ± 3.65	24.01 ± 4.57	25.07 ± 2.24	1.318	0.270
2-6	22.97 ± 3.35	21.61 ± 4.27	22.73 ± 2.17	1.873	0.157
6-10	24.76 ± 4.97	22.99 ± 5.41	24.09 ± 4.84	1.717	0.183
10-12	34.91 ± 7.02	32.85 ± 7.85	35.27 ± 7.87	1.048	0.353
Total	25.83 ± 3.67	24.26 ± 4.60	25.55 ± 3.00	2.158	0.119
Postop					
0-2	25.94 ± 2.07	25.63 ± 2.85	-	0.616	0.539
2-6	24.51 ± 1.94	24.22 ± 3.00	-	0.458	0.648
6-10	25.34 ± 4.10	24.21 ± 4.27	-	0.255	0.799
10-12	33.34 ± 6.54	31.33 ± 7.23	-	0.572	0.569
Total	26.38 ± 2.45	25.55 ± 3.19	-	0.601	0.549
Preop - Postop					
0-2	0.67 ± 0.49	1.62 ± 1.13	-	1.079	0.284
2-6	1.54 ± 0.45*	2.61 ± 1.09*	-	1.404	0.164
6-10	0.58 ± 0.72	1.22 ± 1.43	-	0.756	0.452
10-12	-1.58 ± 1.08	-1.52 ± 1.97	-	0.031	0.975
Total	0.55 ± 0.51	1.29 ± 1.13	-	0.946	0.346

**Table 3:** Corneal densitometry comparisons of the central corneal layer “Preop-Postop” section indicates magnitude of change from preoperative to postoperative measurements. Comparisons between study groups and control group for “Postop” not available, as control group patients did not undergo surgery. Values represented mean ± standard deviation; Statistical significance for “Preop” section determined with one-way ANOVA (F score) and “Postop” and “Preop-Postop” section determined with two-sample T-test (T-value); set at p<0.05. Units = grayscale units (GSU) * Significantly higher than corneal densitometry of control group only (p<0.05)

Annulus (mm)	Non-haze group (N=81)	Transient haze group (N=17)	Controls (N=78)	F score/T-value	P-value
Preop					
0-2	15.75 ± 1.55	15.44 ± 1.97	15.51 ± 1.16	0.583	0.559
2-6	14.54 ± 1.51	14.05 ± 1.88	14.13 ± 1.17	2.047	0.132
6-10	17.31 ± 3.77* (p=0.037, t=2.314)	16.25 ± 3.70	16.18 ± 3.92	2.880	0.059
10-12	23.99 ± 4.69	22.92 ± 3.99	23.56 ± 4.89	0.684	0.506
Total	17.17 ± 2.48	16.43 ± 2.58	16.53 ± 2.20	2.641	0.074
Postop					
0-2	15.68 ± 1.28	15.92 ± 1.41	-	1.171	0.244
2-6	14.78 ± 1.21	14.72 ± 1.25	-	0.446	0.656
6-10	17.74 ± 3.73	16.76 ± 2.86	-	0.027	0.978
10-12	23.54 ± 4.24	22.49 ± 3.41	-	0.141	0.888
Total	17.28 ± 2.15	16.81 ± 1.71	-	0.135	0.893
Preop-Postop					
0-2	-0.07 ± 0.22	0.48 ± 0.52	-	1.183	0.240
2-6	0.25 ± 0.21	0.67 ± 0.49	-	1.034	0.304
6-10	0.43 ± 0.53	0.51 ± 0.98	-	0.150	0.881
10-12	-0.45 ± 0.64	-0.43 ± 1.10	-	0.058	0.954
Total	0.11 ± 0.33	0.38 ± 0.66	-	0.615	0.540

**Table 4:** Corneal densitometry comparisons of the posterior corneal layer “Preop-Postop” section indicates magnitude of change from preoperative to postoperative measurements. Comparisons between study groups and control group for “Postop” not available, as control group patients did not undergo surgery. Statistical significance for “Preop” section determined with one-way ANOVA (F score) and “Postop” and “Preop-Postop” section determined with two-sample T-test (T-value); set at p<0.05. Values represented mean ± standard deviation; Units = grayscale units (GSU) * Indicates statistically significant increase in corneal densitometry from “Preop” to “Postop” within the specified group (p<0.05).

Annulus (mm)	Non-haze group (N=81)	Transient haze group (N=17)	Controls (N=78)	F score/T-value	P-value
Preop					
0-2	11.89 ± 2.10	11.63 ± 2.58	11.92 ± 1.49	0.185	0.831
2-6	11.36 ± 2.04	10.98 ± 2.35	11.21 ± 1.42	0.400	0.671
6-10	14.89 ± 3.86	13.82 ± 3.28	14.39 ± 3.41	1.365	0.258
10-12	20.36 ± 5.03	18.83 ± 3.64	19.67 ± 4.98	1.262	0.286
Total	14.06 ± 2.89	13.30 ± 2.76	13.73 ± 2.26	1.099	0.335
Postop					
0-2	11.96 ± 1.32	12.05 ± 1.56	-	0.489	0.626
2-6	12.00 ± 1.28	12.00 ± 1.57	-	0.405	0.686
6-10	16.08 ± 3.47	15.34 ± 2.80	-	0.048	0.962
10-12	21.24 ± 4.59	20.11 ± 3.47	-	0.116	0.908
Total	14.80 ± 2.17	14.37 ± 2.04	-	0.069	0.945
Preop - Postop					
0-2	0.07± 2.29	0.43 ± 0.62	-	0.764	0.447
2-6	0.65 ± 2.28*	1.02 ± 0.59	-	0.921	0.359
6-10	1.19 ± 0.55*	1.52 ± 0.90	-	0.722	0.472
10-12	0.88 ± 0.69	1.29 ± 0.99	-	0.695	0.489
Total	0.73 ± 0.39	1.08 ± 0.71	-	0.789	0.432

**Table 5:** Corneal densitometry comparisons of the full thickness corneal layer “Preop-Postop” section indicates magnitude of change from preoperative to postoperative measurements. Comparisons between study groups and control group for “Postop” not available, as control group patients did not undergo surgery. Statistical significance for “Preop” section determined with one-way ANOVA (F score) and “Postop” and “Preop-Postop” section determined with two-sample T-test (T-value); set at p<0.05. Values represented mean ± standard deviation; Units = grayscale units (GSU) * Indicates statistically significant increase in corneal densitometry from “Preop” to “Postop” within the specified group (p<0.05).

Annulus (mm)	Non-haze group (N=81)	Transient haze group (N=17)	Controls (N=78)	F score/T-value	P-value
Preop					
0-2	17.63 ± 2.25	17.02 ± 2.95	17.50 ± 1.51	0.712	0.492
2-6	16.29 ± 2.13	15.55 ± 2.72	16.02 ± 1.48	1.378	0.255
6-10	18.99 ± 4.05	17.68 ± 3.96	18.23 ± 3.95	1.951	0.145
10-12	26.42 ± 4.99	24.86 ± 4.71	26.17 ± 5.48	1.059	0.349
Total	19.03 ± 2.84	18.01 ± 3.20	18.61 ± 2.37	1.917	0.150
Postop					
0-2	17.85 ± 1.37	17.86 ± 1.63	-	0.192	0.848
2-6	17.01 ±1.34	16.99 ±1.70	-	0.040	0.968
6-10	19.72 ± 3.68	18.77 ± 3.11	-	0.082	0.934
10-12	26.04 ± 4.58	26.64 ± 4.21	-	0.363	0.717
Total	19.49 ± 2.13	18.92 ± 2.10	-	0.165	0.869
Preop - Postop					
0-2	0.22 ± 0.31	0.84 ± 0.71	-	1.120	0.265
2-6	0.72 ± 0.29*	1.44 ± 0.68*	-	1.303	0.196
6-10	0.73 ± 0.58	1.09 ± 1.06	-	0.636	0.526
10-12	-0.38 ± 0.71	1.78 ± 1.21	-	0.235	0.815
Total	0.46 ± 0.39	0.91 ± 0.79	-	0.862	0.391

One-year postoperative comparisons of densitometry

Across all corneal layers and annuli, there were no significant differences in postoperative CD between the non-haze and transient haze groups (p>0.05). Additionally, a comparison of the change in CD within the non-haze and transient haze groups was conducted.

**Figure 1:**
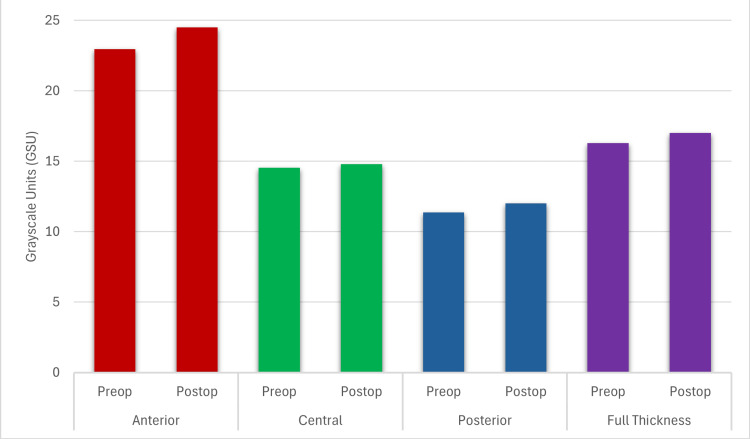
Preop vs. postop corneal densitometry values in the 2–6 mm annulus of the non-haze group Statistically significant difference between preop and postop in the anterior, posterior, and full thickness layers in the non-haze group. Statistical significance set at p<0.05.

**Figure 2:**
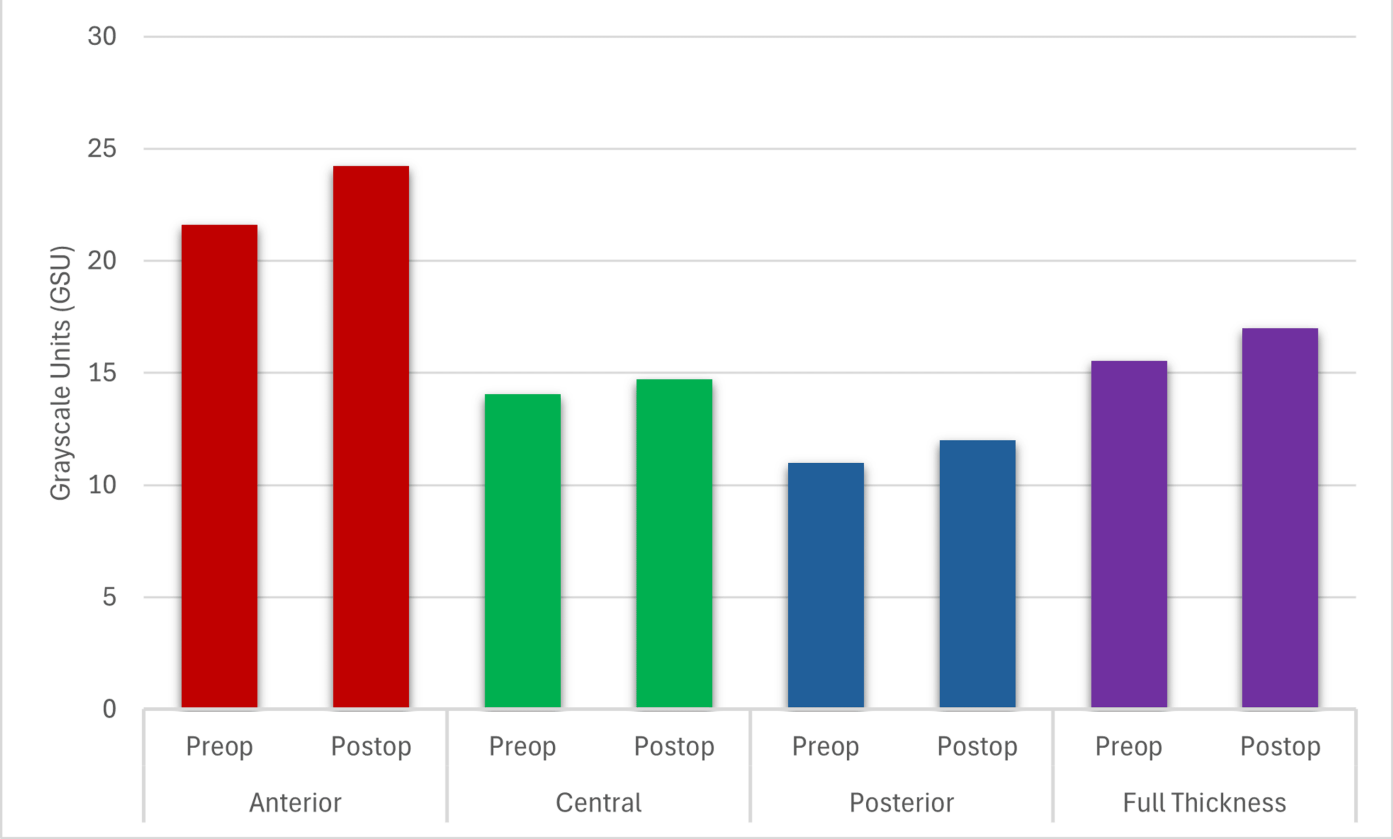
Preop vs. postop corneal densitometry values in the 2–6 mm annulus of the transient haze group Statistically significant difference between preop and postop in the anterior and full thickness layers in the transient haze group. Statistical significance set at p<0.05.

In the non-haze group, postoperative CD increased compared to preoperative values in the 2-6 mm annulus of the anterior (1.54 ± 0.45 GSU; p=0.001), posterior (0.65 ± 2.28 GSU; p=0.032), and full-thickness (0.72 ± 0.29 GSU; p=0.006) corneal layers, as well as in the 6-10 mm annulus of the posterior corneal layer (1.19 ± 0.55 GSU; p=0.049). Similarly, in the transient haze group, an increase in CD was observed in the 2-6 mm annulus of the anterior (2.61 ± 1.09 GSU; p=0.021) and full-thickness (1.44 ± 0.68 GSU; p=0.039) corneal layers (Tables 2-5; Figures 1, 2).

## Discussion

The transient subepithelial opacification of the cornea following refractive surgeries, including PRK, LASIK, and SMILE, commonly referred to as corneal “haze,” has traditionally been diagnosed using clinical grading scales such as the one proposed by Fantes et al. [9]. However, due to the subjective nature of these tools, an objective predictor of postoperative haze has long been sought after [14]. CD, which quantifies corneal backscatter, has been proposed as a potential predictor of haze [13,15-18]. While previous studies have evaluated the efficacy of CD as a predictor of haze in PRK, to our knowledge, this study is the first to assess lasting changes in CD in patients with a history of postoperative haze following SMILE surgery [13].

Preoperatively, SMILE patients were younger, and had more myopic spheres, less cylindrical astigmatism, more myopic SEQ, and thicker corneas compared to the control group. Otherwise, there were no significant differences in sex, K1, or K2. This makes sense because SMILE is often reserved for high myopes with lower cylinder values and thicker corneas [19]. However, preoperative densitometry measurements were nearly identical among the non-haze, transient haze, and control groups. This supports the assumption that the SMILE cohort in this study was not significantly different from the general population, aside from refractive error.

Regarding the postoperative rate of haze development, our study found an incidence of 17% among the 98 eyes that underwent SMILE. This is higher than the 8% incidence reported by Ivarsen et al. in a larger study of 1,800 eyes [20]. The discrepancy may be partially explained by our smaller sample size. Furthermore, since haze is traditionally diagnosed clinically using grading scales, the interpretation of these classifications can vary among physicians, potentially leading to differences in the thresholds for diagnosis. Another reason for the observed differences in the postoperative rate of haze development could be the follow-up time; we followed patients for up to one year after surgery, while Ivarsen et al. evaluated their patients only after three months.

At one year, there were no significant differences in CD between the non-haze and transient haze groups. When evaluating changes in densitometry before and after SMILE, both groups showed a significant increase only in the 2-6 mm annulus of the anterior corneal and full-thickness corneal layers. These findings are consistent with previous studies that demonstrated minimal differences in postoperative densitometry except in the 2-6 mm annulus [21,22]. Thus, our results suggest that an increase in CD in the 2-6 mm annulus is the only lasting change in CD in patients who underwent SMILE.

The overall lack of differences in CD noted between SMILE patients who developed haze and those who did not may be explained by our measurement of CD at one year postoperatively, at which time corneal haze had resolved in all but two of the eyes included in our study. Previous studies that were able to identify changes in CD related to corneal haze in PRK patients did so by measuring CD at earlier postoperative visits (one month, three months, etc.) in patients actively experiencing haze [13]. To make conclusions regarding the relationship between CD and active corneal haze in SMILE patients, future studies should obtain CD measurements at earlier postoperative periods. Other limitations of our study included the retrospective nature of the study, the sample size of patients who developed haze, and potential self-selection bias of patients who chose to follow-up at the one-year postoperative period. Despite these limitations, this is one of the few studies to assess for lasting changes in CD in patients who developed postoperative haze after undergoing SMILE.

## Conclusions

Our study found no difference in CD postoperatively between patients who developed haze after SMILE and those who did not. Both groups experienced an increase in CD in the 2-6 mm annulus of the anterior cornea following SMILE, regardless of haze status, with no significant difference in the magnitude of this increase. Although our study did not directly measure CD in patients actively experiencing haze, it can be concluded that there is no lasting difference in CD between those who develop haze and those who do not. Further research with more frequent CD measurements in patients actively experiencing haze is needed to better understand the relationship between CD and haze in SMILE patients.
